# Effect of Vitamin C on Clinical Outcomes of Critically Ill Patients With COVID-19: An Observational Study and Subsequent Meta-Analysis

**DOI:** 10.3389/fmed.2022.814587

**Published:** 2022-02-11

**Authors:** Evdokia Gavrielatou, Eleni Xourgia, Nikoleta A. Xixi, Athina G. Mantelou, Eleni Ischaki, Aggeliki Kanavou, Dimitris Zervakis, Christina Routsi, Anastasia Kotanidou, Ilias I. Siempos

**Affiliations:** ^1^First Department of Critical Care Medicine and Pulmonary Services, Evangelismos Hospital, Medical School, National and Kapodistrian University of Athens, Athens, Greece; ^2^Division of Pulmonary and Critical Care Medicine, Department of Medicine, New York-Presbyterian Hospital, Weill Cornell Medical Center, Weill Cornell Medicine, New York, NY, United States

**Keywords:** acute respiratory distress syndrome, acute respiratory failure, pneumonia, mechanical ventilation, intensive care unit, coronavirus

## Abstract

**Background:**

Whether vitamin C provides any benefit when administered in critically ill patients, including those with coronavirus disease (COVID-19), is controversial. We endeavored to estimate the effect of administration of vitamin C on clinical outcomes of critically ill patients with COVID-19 by performing an observational study and subsequent meta-analysis.

**Methods:**

Firstly, we conducted an observational study of critically ill patients with laboratory-confirmed COVID-19 who consecutively underwent invasive mechanical ventilation in an academic intensive care unit (ICU) during the second pandemic wave. We compared all-cause mortality of patients receiving vitamin C (“vitamin C” group) or not (“control” group) on top of standard-of-care. Subsequently, we systematically searched PubMed and CENTRAL for relevant studies, which reported on all-cause mortality (primary outcome) and/or morbidity of critically ill patients with COVID-19 receiving vitamin C or not treatment. Pooled risk ratio (RR) and 95% confidence intervals (CI) were calculated using a random effects model. The meta-analysis was registered with PROSPERO.

**Results:**

In the observational study, baseline characteristics were comparable between the two groups. Mortality was 20.0% (2/10) in the vitamin C group vs. 47.6% (49/103; *p* = 0.11) in the control group. Subsequently, the meta-analysis included 11 studies (6 observational; five randomized controlled trials) enrolling 1,807 critically ill patients with COVID-19. Mortality of patients receiving vitamin C on top of standard-of-care was not lower than patients receiving standard-of-care alone (25.8 vs. 34.7%; RR 0.85, 95% CI 0.57–1.26; *p* = 0.42).

**Conclusions:**

After combining results of our observational cohort with those of relevant studies into a meta-analysis of data from 1,807 patients, we found that administration vitamin C as opposed to standard-of-care alone might not be associated with lower of mortality among critically ill patients with COVID-19. Additional evidence is anticipated from relevant large randomized controlled trials which are currently underway.

**Systematic Review Registration:**

https://www.crd.york.ac.uk/prospero/, identifier: CRD42021276655.

## Background

In 2017, a retrospective before-and-after study showed a significant improvement in the survival of critically ill septic patients who received vitamin C (combined with hydrocortisone and thiamine) (1). Subsequently, several large randomized controlled trials explored the effect of this intervention on clinical outcomes of patients with severe sepsis, yielding contradicting results (2–6). By combining results of the above trials, a recent meta-analysis concluded that administration of vitamin C was associated with shorter duration of vasopressor use (albeit not with lower mortality) in such patients (7). Taken together, current evidence does not seem to preclude a potentially beneficial effect of administration of intravenous high-dose vitamin C on clinical outcomes of critically ill patients, at least those with sepsis.

The hypothesis that vitamin C may be beneficial in critically ill patients seems to be based on sound biological rationale. Indeed, vitamin C is a potent antioxidant, it affects inflammation and vascular integrity, while it serves as an enzyme cofactor essential for synthesis of endogenous catecholamines (8, 9). Such biological functions might justify the hypothesis that administration of vitamin C might be beneficial in patients with coronavirus disease (COVID-19) as well, at least those with critical illness (10, 11). Nevertheless, only a few studies explored the effect of this intervention on outcomes of critically ill patients with COVID-19 and those studies have not yet been systematically synthesized. Therefore, the COVID-19 Treatment Guidelines, issued by the National Institutes of Health, stated that “there is insufficient evidence to recommend either for or against the use of vitamin C for the treatment of COVID-19 in critically ill patients” (12).

Given both the interest and limited evidence on this issue, we designed a combined study. Firstly, we carried out an observational study exploring the effect of administration of vitamin C on clinical outcomes of critically ill patients with COVID-19 who consecutively underwent invasive mechanical ventilation in an academic intensive care unit (ICU). Then, we combined our results with those of relevant studies in a systematic review and meta-analysis.

## Methods

The present work consisted of two components: an “observational study” and a subsequent “meta-analysis,” in which results of the observational study were combined with results of relevant studies.

### Observational Study

#### Study Design

We conducted an observational retrospective cohort study including adult (>18 years old) patients with polymerase chain reaction (PCR)-confirmed Severe Acute Respiratory Syndrome Coronavirus-2 (SARS-CoV-2) infection who consecutively underwent invasive mechanical ventilation in an academic ICU of a tertiary hospital (Evangelismos Hospital, Athens, Greece) during the second pandemic wave (specifically, between October 21st, 2020 and March 8th, 2021). The Institutional Review Board at Evangelismos Hospital (116/31-03-2021) approved of the data collection and waived the need of informed consent. We followed the “Strengthening the Reporting of Observational Studies in Epidemiology” (STROBE) statement guidelines (Supplementary Material).

#### Compared Groups and Data Collection

All patients underwent invasive mechanical ventilation due to hypoxemia; i.e., they met the Berlin criteria of acute respiratory distress syndrome (ARDS). All patients were administered dexamethasone (6 mg/day intravenously for at least 5 days) as part of their standard-of-care treatment of critical COVID-19 (13). Based on clinical judgment of their treating ICU clinicians and following national standard operating procedures for the administration of “off-label” medications, several patients received vitamin C on top of standard-of-care treatment and those patients comprised the “vitamin C” group. Specifically, within the first 24 h from their intubation, those patients received intravenously 1 g vitamin C plus thiamine 500 mg every 8 h for 4 days; then, intravenously 500 mg vitamin C plus thiamine 250 mg every 8 h for 3 days; and, finally, intravenously 500 mg vitamin C plus thiamine 250 mg every 12 h for 3 days. Therefore, they received intravenous high-dose vitamin C (Pabrinex®, Kyowa Kirin Limited, United Kingdom) for a total of 10 days or less (in case of ICU discharge or death). On the other hand, patients who received only standard-of-care treatment comprised the “control” group. This observational retrospective study took advantage of the fact that attending ICU clinicians gave vitamin C in some (“vitamin C” group) but not all (“control” group) patients.

In addition to data on administration or not of vitamin C, we gathered data on demographics, comorbidities, respiratory support (including high-flow nasal oxygen) prior to intubation along with ventilator settings, lung mechanics and Sequential Organ Failure Assessment (SOFA) score on day of intubation. The respiratory component of SOFA was calculated after the intubation, while the remaining SOFA components (namely, coagulation, hepatic, cardiovascular, neurologic and renal) were calculated prior to intubation.

#### Outcomes

We considered all-cause ICU-mortality as the primary outcome of the observational study. Vasopressor-free days, continuous renal replacement-free days, ventilator-free days and ICU-free days were the secondary outcomes. As previously (14, 15), we calculated vasopressor-free days, continuous renal replacement therapy-free days, ventilator-free days and ICU-free days by the number of days in the first 28 days following intubation that a patient was alive and not receiving vasopressors, not receiving continuous renal replacement therapy, not on a ventilator or not in the ICU, respectively. We censored outcomes at day 28 following intubation.

### Meta-Analysis

Subsequently, we carried out a systematic review and meta-analysis of relevant studies in accordance with the Preferred Reporting Items for Systematic Reviews and Meta-Analyses (PRISMA) statement (16). We prespecified search strategy, data extraction and outcomes in a protocol registered with PROSPERO (CRD42021276655) and available online.

#### Search Strategy

Two authors (EX and NAX) independently conducted the literature search. In addition to PubMed and CENTRAL, we systematically searched preprint servers (namely, medRxiv and Research Square) to capture rapidly accumulated evidence, as previously done (17). We used Boolean logic to create the search phrase: (“ascorbic” OR “vitamin C” OR “vit C”) AND (“coronavirus” OR “COVID” OR “COVID 19” OR “SARS-CoV2”). We retrieved relevant literature up to December 18th, 2021, with no language restrictions. We considered for inclusion observational studies and randomized controlled trials, which compared administration of vitamin C on top of standard-of-care vs. standard-of-care alone in critically ill patients with COVID-19 and reported data on all-cause mortality and/or morbidity. Case reports and case series involving less than 5 patients were excluded.

#### Data Extraction and Risk of Bias Assessment

Two authors (EX and NAX) independently extracted data in a prespecified worksheet and cross-checked their findings. For each included study, we collected data on author, country, study design, number of critically ill patients with COVID-19 receiving or not vitamin C, administered regimen of vitamin C, patient characteristics (i.e., demographics and comorbidities, such as diabetes mellitus, hypertension, ischemic cardiac disease) and outcomes.

Two authors (EX and NAX) independently assessed the risk of bias of included studies. Any disagreements were discussed with the corresponding author (IIS). For assessment of observational studies, we used the Tool to Assess Risk of Bias in Cohort Studies, developed by the CLARITY Group at McMaster University (18). For assessment of randomized controlled trials, we used the Risk of Bias 2 (RoB 2) tool (19). We provided details on the risk of bias assessment in the Supplementary Material.

#### Outcomes

We considered all-cause mortality as the primary outcome of the meta-analysis. Length of ICU stay, duration of mechanical ventilation, need for renal replacement therapy and adverse events related to vitamin C were the secondary outcomes.

### Statistical Analyses

For the observational study, we used SPSS software 22.0 (IBM, Armonk, NY, USA). We presented continuous variables as median and interquartile range (IQR) and compared them using Mann-Whitney rank sum test. We presented categorical variables as number of patients (percentage) and compared them using chi-squared or Fisher's exact test. We performed a complete case analysis because missing data on outcomes were below 3% and completely at random according to Little's MCAR test (20). All statistical tests were 2-tailed.

For the meta-analysis, we used Review Manager 5.4 (RevMan 5.4.1, Cochrane Collaboration) (21). We expressed pooled dichotomous effect measures and pooled continuous effect measures as risk ratio (RR) with 95% confidence intervals (CI) and mean difference (MD) with 95% CI, respectively. We transformed continuous values presented as median to mean (22). We conservatively utilized a random effects model. We assessed the presence of statistical heterogeneity with *I*^2^, interpreted according to the Cochrane Handbook recommendations; 0–40%: might not be important; 30–60%: may represent moderate heterogeneity; 50–90%: substantial heterogeneity; 75–100%: considerable heterogeneity (22). We carried out two pre-specified sensitivity analyses by including only (a) studies with low risk of bias; and (b) randomized controlled trials. We considered a *p* < 0.05 to denote statistical significance.

## Results

### Observational Study

During the study period, 113 patients [24.8% female, median age 69.0 (IQR 57.0–76.5) years] consecutively underwent invasive mechanical ventilation in the ICU and were therefore included in the observational study. Ten patients (8.8% of the cohort) received intravenous high-dose vitamin C on top of standard-of-care (vitamin C group), while the rest received only standard-of-care (control group). Table 1 shows that baseline characteristics of included patients, such as demographics, comorbidities, SOFA score and lung mechanics, were comparable between the two groups. At baseline, partial pressure of arterial oxygen to fraction of inspired oxygen ratio (PaO_2_:FiO_2_) was lower in the vitamin C than control group [95.4 (68.3–145.8) vs. 142.5 (113.3–182.5); *p* = 0.012].

**Table 1:** Baseline characteristics of patients included in the observational study.

**Characteristic**	**Vitamin C group (*n* = 10)**	**Control group (*n* = 103)**	***p*-value**
Age, years	70.5 (58.0–75.0)	69.0 (55.0–77.0)	0.927
Female sex	3 (30.0)	25 (24.3)	0.709
Race			1.000
Caucasian	10 (100.0)	100 (97.1)	
Asian/Middle Eastern	0 (0.0)	2 (1.9)	
African	0 (0.0)	1 (1.0)	
Comorbidity	7 (70.0)	77 (74.8)	0.715
Chronic kidney disease	2 (20.0)	13 (12.6)	0.620
Chronic lung disease	1 (10.0)	16 (15.5)	1.000
Heart condition	3 (30.0)	25 (24.3)	0.707
Hypertension	6 (60.0)	55 (53.4)	0.751
Liver disease	0 (0.0)	0 (0.0)	-
Diabetes mellitus	3 (30.0)	23 (22.3)	0.694
Malignancy	0 (0.0)	9 (8.7)	1.000
SOFA score on the day of intubation	4.0 (4.0–5.3)	4.0 (4.0–5.0)	0.743
Respiratory	4.0 (4.0–4.0)	4.0 (4.0–4.0)	0.139
Coagulation	0.0 (0.0–0.3)	0.0 (0.0–0.0)	0.708
Hepatic	0.0 (0.0–0.0)	0.0 (0.0–0.0)	0.363
Cardiovascular	0.0 (0.0–0.0)	0.0 (0.0–0.0)	0.363
Neurologic	0.0 (0.0–0.0)	0.0 (0.0–0.0)	0.397
Renal	0.0 (0.0–1.0)	0.0 (0.0–0.0)	0.587
Days from symptom onset to intubation	5.5 (3.8–9.3)	7.0 (4.0–11.0)	0.314
Usage of high-flow nasal oxygen	8 (80.0)	69 (67.0)	0.499
Duration of high-flow nasal oxygen, days	1.5 (1.0–5.8)	2.0 (1.0–4.5)	0.930
Usage of non-rebreather mask	0.0 (0.0)	22 (21.4)	0.205
Duration of non-rebreather mask, days	NA	2.0 (1.0–3.0)	NA
**Lung mechanics on the day of intubation**			
Ventilation mode			0.013
Volume Control	10 (100.0)	61 (59.2)	
Pressure Control	0.0 (0.0)	42 (40.8)	
Respiratory rate, bpm	25.0 (22.0–30.0)	25.0 (22.0–28.0)	0.722
Tidal volume, mL	475.0 (442.5–500.0)	480.0 (440.0–490.0)	0.775
PEEPext, cmH_2_O	10.5 (10.0–14.0)	12.0 (10.0–13.0)	0.857
PEEPtotal, cmH_2_O	10.5 (10.0–14.0)	13.0 (10.0–15.0)	0.237
Pplateau, cmH_2_O	24.5 (22.3–27.8)	25.0 (23.0–27.8)	0.760
Pdriving, cmH_2_O	13.0 (13.0–14.8)	12.0 (10.0–14.3)	0.223
FiO_2_	1.0 (0.9–1.0)	0.9 (0.7–1.0)	0.052
PaO_2_, mmHg	76.5 (68.3–145.8)	110.0 (95.0–140.0)	0.040
PaO_2_:FiO_2_	95.4 (68.3–145.8)	142.5 (113.3–182.5)	0.012
PaCO_2_, mmHg	50.5 (42.9–58.5)	47.0 (41.0–56.0)	0.511

*n, number; NA, not applicable; SOFA, sequential organ failure assessment; bpm, breaths per minute; PEEP, positive end expiratory pressure; Pplateau, plateau pressure; Pdriving, driving pressure; PaO_2_, partial pressure of arterial oxygen; FiO_2_, fraction of inspired oxygen; PaCO_2_, partial pressure of arterial carbon dioxide. Data are presented as median (interquartile range) or number (%)*.

*Heart condition included congestive heart failure, coronary artery disease, and cardiomyopathies*.

Table 2 summarizes outcomes of included patients. All-cause ICU-mortality was 20.0% (2/10) in the vitamin C group vs. 47.6% (49/103; *p* = 0.110) in the control group. There was no difference between the vitamin C and control group in terms of vasopressor-free days (9.0 vs. 0.0, *p* = 0.271), continuous renal replacement therapy-free days (26.0 vs. 19.0; *p* = 0.644), ventilator-free days (0.0 vs. 0.0; *p* = 0.832) or ICU-free days (0.0 vs. 0.0; *p* = 0.667).

**Table 2:** Outcomes of patients included in the observational study.

**Outcome**	**Vitamin C group (*n* = 10)**	**Control group (*n* = 103)**	***p*-value**
Vasopressor-free days, days	9.0 (0.0–24.0)	0.0 (0.0–14.0)	0.271
Continuous renal replacement therapy-free days, days	26.0 (6.8–28.0)	19.0 (5.8–28.0)	0.644
Ventilator-free days, days	0.0 (0.0–18.5)	0.0 (0.0–15.0)	0.832
ICU-free days, days	0.0 (0.0–3.3)	0.0 (0.0–8.0)	0.667
ICU-mortality	2 (20.0)	49 (47.6)	0.110

*n, number; ICU, intensive care unit*.

*Data are presented as median (interquartile range) or number (%)*.

*Vasopressor-free days, continuous renal replacement therapy-free days, ventilator-free days and ICU-free days were calculated by the number of days in the first 28 days following intubation that a patient was alive and not receiving vasopressors, not receiving continuous renal replacement therapy, not on a ventilator or not in the ICU, respectively. All outcomes were censored at day 28 following intubation*.

### Meta-Analysis

**Figure 1:**
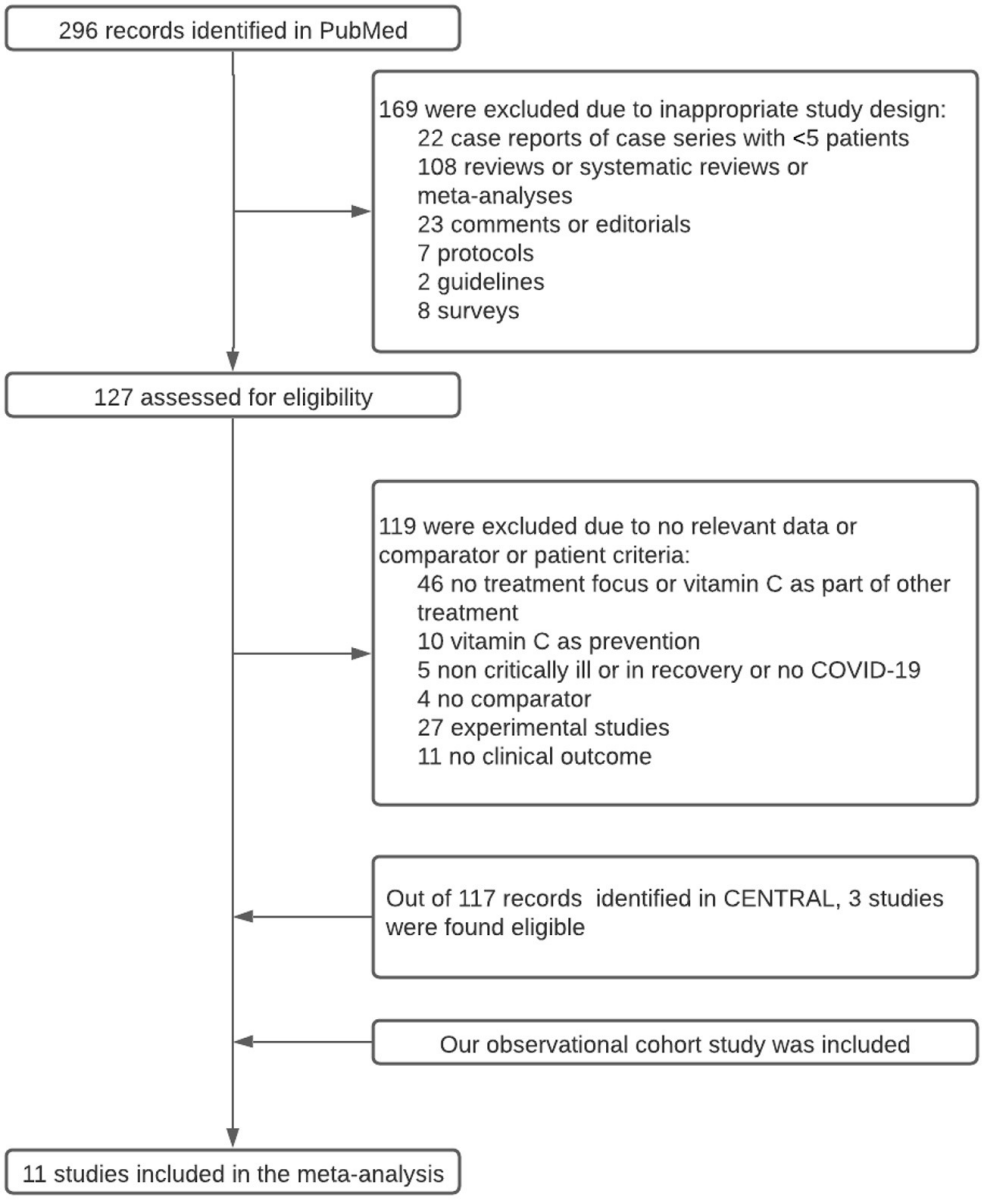
Study flow diagram.

Figure 1 shows the flow diagram for study selection. Out of the 413 initially retrieved articles, 11 studies [i.e., 10 studies (23–32) from the literature plus our observational study], involving a total of 1,807 critically ill patients (515 received vitamin C) with COVID-19, were incorporated in the meta-analysis. Table 3 summarizes characteristics of the included studies. Six of them were retrospective observational studies (23, 24, 26, 28, 30) and five were randomized controlled trials (25, 27, 29, 31, 32). Supplementary Table 1 summarizes risk of bias assessment of the included studies. Six (23, 25, 28–30, 32) of them were considered to have low risk of bias.

**Table 3:** Characteristics of individual studies included in the meta-analysis comparing vitamin C vs. control.

**Author/Country**	**Study design**	**Administered vitamin C regimen**	**Number of critically ill patients (n)**	**Female sex (%)**	**Age (years)**	**Baseline severity**	**Hypertension (%)**	**Diabetes Mellitus (%)**	**Coronary heart disease (%)**
Al Sulaiman/Saudi Arabia (23)	Observational retrospective, multi-center	Enterally 1 g q24h	739	15.2 vs. 30.0	60.5 ± 15.1 vs. 60.7 ± 14.8	4.0 (2.0–6.0) vs. 5.0 (3.0–8.0)	56.4 vs. 56.9	60.3 vs. 61.2	8.2 vs. 8.6
Beigmohammadi/Iran (32)	Randomized controlled trial, single-center	Enterally 2 g q24h	60	50.0 vs. 46.7	51.0 ± 17.3 vs. 53.0 ± 7.0	7.0 ± 2.3 vs. 7.0 ± 3.0	NA	NA	NA
Darban/Iran (31)	Observational retrospective, single-center	IV 2 g q6h	20	NA	NA	NA	NA	NA	NA
Gao^*^/China (24)	Observational retrospective, single-center	IV 6 g q12h on 1st day; then, IV 6 g q24h for the next 4 days	76	54.3 vs. 53.3	63.0 (54.0–71.0) vs. 57.0 (49.0–67.0)	NA	34.8 vs. 20.0	23.9 vs. 13.3	6.5 vs. 6.7
Gavrielatou/Greece	Observational retrospective, single-center	IV 1 g q8h for 4 days; then, IV 500 mg q8h for 3 days; and, finally, IV 500 mg q12h for 3 days	113	30.0 vs. 24.3	70.5 (58.0–75.0) vs. 69.0 (55.0–77.0)	4.0 (4.0–5.3) vs. 4.0 (4.0–5.0)	60.0 vs. 53.4	30.0 vs. 22.3	30.0 vs. 24.3^**^
JamaliMoghadamSiahkali/ Iran (25)	Randomized controlled trial, single-center	IV 1.5 g q6h for 5 days	60	50.0 vs. 50.0	57.5 ± 18.2 vs. 61 ± 15.9	3.6 ± 1.4 vs. 3.4 ± 1.5	50.0 vs. 33.3	40.0 vs. 36.7	13.3 vs. 23.3
Krishnan/United States (26)	Observational retrospective, multi-center	NA	152	NA	NA	NA	NA	NA	NA
Kumari/Pakistan (27)	Randomized controlled trial, single-center	IV 50 mg/kg q24h	150	NA	53 ± 11 vs. 53 ± 12	NA	NA	NA	NA
Li/United States (28)	Observational retrospective, single-center	IV 1.5 g q6h for up to 4 days	32	63.0 vs. 63.0	64.1 ± 8.3 vs. 64.9 ± 11.8	6.6 ± 3.5 vs. 9.4 ± 3.2	75.0 vs. 54.2	50.0 vs. 45.8	12.5 vs. 4.2
Zhang/China (29)	Randomized controlled trial, multi-center	IV 12 g q12h for 7 days	56	44.4 vs. 24.1	66.3 ± 11.2 vs. 67 ± 14.3	14.0 (11.0–16.0) vs. 13.0 (9.5–15.0)	37.0 vs. 51.7	29.6 vs. 32.1	14.8 vs. 27.6
Zheng/China (30)	Observational retrospective, single-center	IV 2–4 g q24 24h after admission or during follow up before discharge	397	40.0 vs. 49.5	67.5 (58.0–74.8) vs. 67.0 (62.0–74.0)	NA	18.6 vs. 21.4	15.7 vs. 15.6	4.3 vs. 6.7

*n, number; IV, intravenous; h, hours; NA, not applicable*.

*Data are presented as mean ± standard deviation or median (interquartile range)*.

*Baseline severity is presented as Sequential Organ Failure Assessment Score (SOFA) (23, 28) as Acute Physiology And Chronic Health Evaluation (APACHE II) (29) or other (25, 32)*.

* *Data from the entire cohort of patients (not only critically ill) are presented*.

** *Numbers include coronary heart disease along with congestive heart failure and cardiomyopathies*.

**Figure 2:**
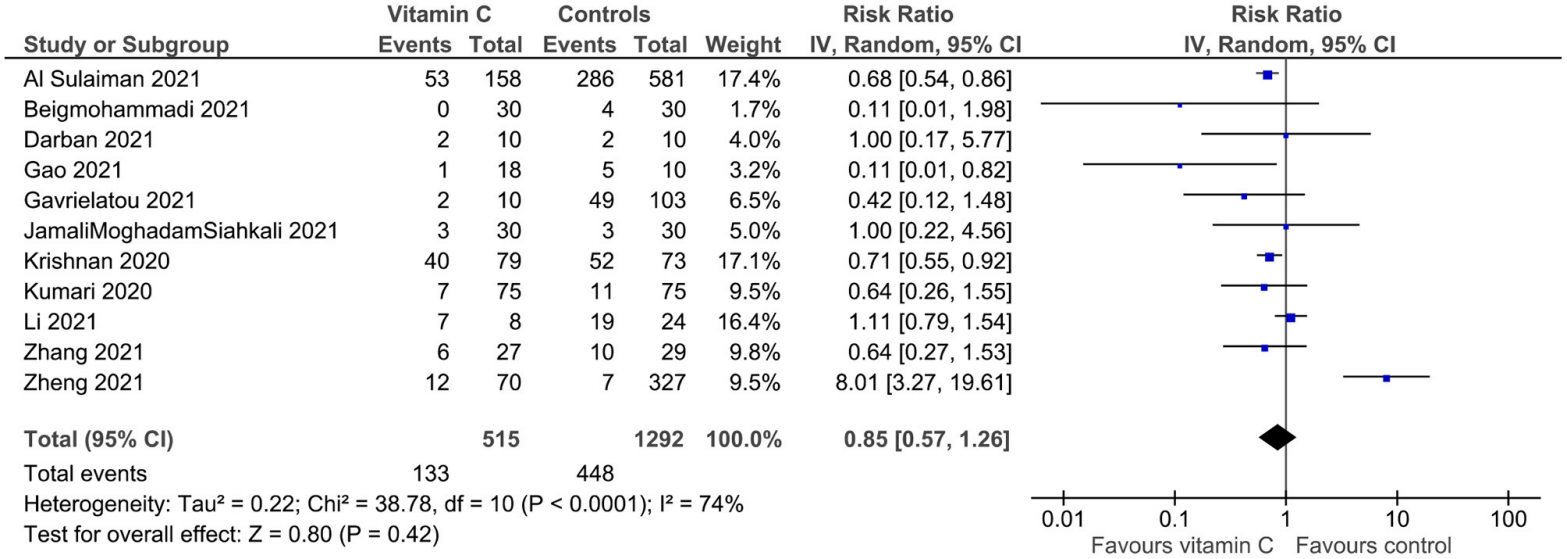
All-cause mortality of critically ill patients with COVID-19 receiving vitamin C on top of standard-of-care (vitamin C group) vs. standard-of-care alone (control group). Pooled risk ratio (RR) and 95% confidence intervals (CI) were calculated using a random effects model.

All 11 (23–32) studies provided data on all-cause mortality. Statistical heterogeneity was important (*I*^2^ = 74%). Mortality was not lower in the vitamin C than control group (25.8 vs. 34.7%; RR 0.85, 95% CI 0.57–1.26; *p* = 0.42; 11 studies; 1,807 patients; 561 deaths; Figure 2). In the sensitivity analysis of studies with low risk of bias (23, 25, 28–30, 32), mortality was 25.1% in the vitamin C group and 32.2% in the control group (RR 1.13, 95% CI 0.59–2.16; *p* = 0.72; six studies; 1,344 patients; 410 deaths; Supplementary Figure 1); while, in the sensitivity analysis of randomized controlled trials (25, 27, 29, 31, 32), the relevant numbers were 10.5 and 17.2%, respectively (RR 0.66, 95% CI 0.39–1.14; *p* = 0.14; five studies; 346 patients; 48 deaths; Supplementary Figure 2).

Regarding secondary outcomes of the meta-analysis, ICU length of stay was longer in the vitamin C than control group (MD 1.56 days, 95% CI 0.63–2.49 days; *p* = 0.001; four studies; 887 patients; Supplementary Figure 3). There was no difference between the vitamin C group and control group in terms of duration of mechanical ventilation (MD 0.40 days, 95% CI −1.81–2.60 days, *p* = 0.73; 2 studies; 852 patients; Supplementary Figure 4) or need for renal replacement therapy (21.6 vs. 34.0%; RR 1.27, 95% CI 0.68–2.39; *p* = 0.45; two studies; 169 patients; 53 events; Supplementary Figure 5). Data on adverse events related to vitamin C were not consistently reported in the included studies.

## Discussion

We carried out a combined study (observational cohort and meta-analysis) to elucidate the effect of vitamin C on clinical outcomes of critically ill patients with COVID-19. In the observational study, we found that mortality of patients with COVID-19 who consecutively underwent invasive mechanical ventilation in an academic ICU was 20.0% in the vitamin C group vs. 47.6% in the control group. In the subsequent meta-analysis of data from 1,807 critically ill patients with COVID-19 enrolled in 11 studies (six observational; five randomized controlled trials), including our observational study, we found that mortality was not lower in the vitamin C than control group (25.8 vs. 34.7%; RR 0.85).

The main finding of our observational study was an association between administration of vitamin C on top of standard-of-care, as opposed to standard-of-care alone, and lower (albeit statistically non-significant) mortality (20.0 vs. 47.6%) in critical COVID-19. Consistently, important clinical outcomes other than mortality, namely vasopressor-free days (9.0 vs. 0.0) and continuous renal replacement therapy-free days (26.0 vs. 19.0), were also in favor (albeit statistically non-significant) of the administration of vitamin C. In our study, vitamin C was administered in high dose, intravenously, initiating as early as 24 h following intubation and for a total of 10 days. All these parameters, namely high (vs. low) dose, intravenous (vs. enteral) administration, early (vs. delayed) initiation, and longer (vs. shorter) duration may reportedly increase the likelihood of a benefit of vitamin C when administered in critically ill patients (27–29). We thought that the fact that favorable differences in outcomes (including mortality) did not reach statistical significance might be due to the small sample size of the observational study (involving 113 patients), which made results prone to statistical error type II. The latter could be addressed by performing a meta-analysis.

We indeed performed a meta-analysis. The main finding of our subsequent meta-analysis (involving 1,807 patients) was again a lack of association between administration of vitamin C on top of standard-of-care, as opposed to standard-of-care alone, and mortality (25.8 vs. 34.7%) in critical COVID-19. This was also the case for the sensitivity analysis of 6 studies with low risk of bias (23, 25, 28–30, 32) (25.1 vs. 32.2%; RR 1.13) and for the sensitivity analysis of 5 randomized controlled trials (25, 27, 29, 31, 32) (10.5 vs. 17.2%; RR 0.66). Another finding of the meta-analysis, as depicted in Table 3, was the variability among the included studies in terms of dose, route and duration of administration along with the lack of information regarding timing of initiation of vitamin C. Given that these parameters may influence the effect of this intervention on clinical outcomes (27–29), standardization of the administration of vitamin C may be desirable.

The main finding of our study was in line with that of a recent relevant meta-analysis, which concluded that “no significant benefit was noted with administration of vitamin C in COVID-19” (33). The latter meta-analysis took into consideration only randomized controlled trials involving both patients with severe and patients with non-severe COVID-19 (33). In contrast, our endeavor may be more comprehensive (by taking into consideration both randomized controlled trials and observational studies) and focused (by taking into consideration only critically ill patients). On the other hand, the authors of another recent relevant pragmatic review of the literature concluded that “intravenous vitamin C intervention may improve oxygenation parameters and reduce inflammatory markers” (34). The latter review, albeit detailed, lacked a meta-analytic approach (34). Taken together, our and previous (33, 34) contributions may provide the readers with the whole picture of the potential role of vitamin C in patients with COVID-19.

Our combined study (observational study and subsequent meta-analysis) has limitations. Firstly, our observational study, due to its design, could not rule out the effect of confounders on the examined association between vitamin C and mortality. However, there was no difference between the compared groups (vitamin C vs. control) in terms of known predictors of mortality in COVID-19 (and therefore potential confounders), such as age, sex, comorbidities, SOFA score and lung mechanics at baseline (35). If anything, baseline oxygenation of mechanically ventilated patients was worse in the vitamin C than control group (PaO_2_:FiO_2_ 95.4 vs. 142.5), which could attenuate a potentially beneficial effect of vitamin C on mortality. Secondly, our observational study was relatively small and therefore it could not lead to a definitive answer by itself. Nevertheless, it contributed valuable data for synthesis in a subsequent meta-analysis. Thirdly, our meta-analysis may be limited by its size (enrolling 1,807 critically ill patients) and the fact that included 6 observational studies, which are probably prone to confounding. However, we attempted to address this limitation by performing a sensitivity analysis of the 5 randomized controlled trials. Lastly, the included studies (23–32) in the meta-analysis did not consistently report on adverse events potentially related to administration of vitamin C, such as oxalate nephropathy, hypernatremia and glucometer error. Nevertheless, relevant evidence before the pandemic indicated that high-dose vitamin C may be relatively safe (36).

## Conclusions

After combining results of our observational cohort with those of relevant studies into a meta-analysis of data from 1,807 patients, we found that administration of vitamin C as opposed to standard-of-care alone might not be associated with lower mortality among critically ill patients with COVID-19. Our combined study (observational cohort and meta-analysis) may constitute the most comprehensive effort to-date to clarify the effect of vitamin C on outcomes of critically ill patients with COVID-19. Additional evidence is anticipated from relevant large randomized controlled trials which are currently underway.

## Data Availability Statement

The original contributions presented in the study are included in the article/Supplementary Material, further inquiries can be directed to the corresponding author.

## Author Contributions

EG contributed to study design, collected data, and interpreted data. EX and NX contributed to study design and the execution of the meta-analysis and they wrote the first draft of the manuscript. EX also undertook statistical analyses. AM, EI, and AKa contributed to data collection. DZ contributed to study design and data interpretation. CR and AKo contributed to data interpretation and critically revised the manuscript. IS conceived of the study, designed the study, supervised the data collection and statistical analyses, and is the guarantor, and final responsibility for the decision to submit for publication. All authors read and approved the final manuscript.

## Funding

This study was supported by grants to IS from the Hellenic Thoracic Society (2019) and the Hellenic Foundation for Research and Innovation (HFRI) under the 2nd Call for HFRI Research Projects to support Post-Doctoral Researchers (Project number: 80-1/15.10.2020).

## Conflict of Interest

The authors declare that the research was conducted in the absence of any commercial or financial relationships that could be construed as a potential conflict of interest.

## Publisher's Note

All claims expressed in this article are solely those of the authors and do not necessarily represent those of their affiliated organizations, or those of the publisher, the editors and the reviewers. Any product that may be evaluated in this article, or claim that may be made by its manufacturer, is not guaranteed or endorsed by the publisher.
