# Supplementary material for: Effect of Vitamin C on Clinical Outcomes of Critically Ill Patients With COVID-19: An Observational Study and Subsequent Meta-Analysis
Source: Front Med (Lausanne). 2022 Feb 11;9:814587. doi: 10.3389/fmed.2022.814587 (PMC8873176; doi:10.3389/fmed.2022.814587)

**Supplementary Appendix**

**Title:  Effect of vitamin C on clinical outcomes of critically ill patients with COVID-19: an observational study and subsequent meta-analysis**

**Authors:** Evdokia Gavrielatou, Eleni Xourgia,^*^ Nikoleta A. Xixi,^*^ Athina G. Mantelou, Eleni Ischaki, Aggeliki Kanavou, Dimitris Zervakis, Christina Routsi, Anastasia Kotanidou, Ilias I. Siempos

*These 2 authors contributed equally.

**A. “Strengthening the Reporting of Observational Studies in Epidemiology” (STROBE) checklist**

|  | Item No. | Recommendation | Page  No. |
| --- | --- | --- | --- |
| **Title and abstract** | 1 | (*a*) Indicate the study’s design with a commonly used term in the title or the abstract | 1 |
|  |  | (*b*) Provide in the abstract an informative and balanced summary of what was done and what was found | 2,3 |
| Introduction | | | |
| Background/rationale | 2 | Explain the scientific background and rationale for the investigation being reported | 4,5 |
| Objectives | 3 | State specific objectives, including any prespecified hypotheses | 4,5 |
| Methods | | | |
| Study design | 4 | Present key elements of study design early in the paper | 5 |
| Setting | 5 | Describe the setting, locations, and relevant dates, including periods of recruitment, exposure, follow-up, and data collection | 5,6,7 |
| Participants | 6 | (*a*) *Cohort study*—Give the eligibility criteria, and the sources and methods of selection of participants. Describe methods of follow-up  *Case-control study*—Give the eligibility criteria, and the sources and methods of case ascertainment and control selection. Give the rationale for the choice of cases and controls  *Cross-sectional study*—Give the eligibility criteria, and the sources and methods of selection of participants | 5,6 |
|  |  | (*b*) *Cohort study*—For matched studies, give matching criteria and number of exposed and unexposed  *Case-control study*—For matched studies, give matching criteria and the number of controls per case |  |
| Variables | 7 | Clearly define all outcomes, exposures, predictors, potential confounders, and effect modifiers. Give diagnostic criteria, if applicable | 5,6 |
| Data sources/ measurement | 8* | For each variable of interest, give sources of data and details of methods of assessment (measurement). Describe comparability of assessment methods if there is more than one group | 5,6,7 |
| Bias | 9 | Describe any efforts to address potential sources of bias |  |
| Study size | 10 | Explain how the study size was arrived at |  |

| Quantitative variables | 11 | Explain how quantitative variables were handled in the analyses. If applicable, describe which groupings were chosen and why | 9,10 | |
| --- | --- | --- | --- | --- |
| Statistical methods | 12 | (*a*) Describe all statistical methods, including those used to control for confounding | | 9,10 |
|  |  | (*b*) Describe any methods used to examine subgroups and interactions | |  |
|  |  | (*c*) Explain how missing data were addressed | | 9,10 |
|  |  | (*d*) *Cohort study*—If applicable, explain how loss to follow-up was addressed  *Case-control study*—If applicable, explain how matching of cases and controls was addressed  *Cross-sectional study*—If applicable, describe analytical methods taking account of sampling strategy | |  |
|  |  | (*e*) Describe any sensitivity analyses | |  |
|  |  | **Results** | |  |
| Participants | 13* | (a) Report numbers of individuals at each stage of study—eg numbers potentially eligible, examined for eligibility, confirmed eligible, included in the study, completing follow-up, and analysed | |  |
|  |  | (b) Give reasons for non-participation at each stage | |  |
|  |  | (c) Consider use of a flow diagram | |  |
| Descriptive data | 14* | (a) Give characteristics of study participants (eg demographic, clinical, social) and information on exposures and potential confounders | | 10,21,22,23 |
|  |  | (b) Indicate number of participants with missing data for each variable of interest | |  |
|  |  | (c) *Cohort study*—Summarise follow-up time (eg, average and total amount) | |  |
| Outcome data | 15* | *Cohort study*—Report numbers of outcome events or summary measures over time | | 10,21,22,23 |
|  |  | *Case-control study—*Report numbers in each exposure category, or summary measures of exposure | |  |
|  |  | *Cross-sectional study—*Report numbers of outcome events or summary measures | |  |
| Main results | 16 | (*a*) Give unadjusted estimates and, if applicable, confounder-adjusted estimates and their precision (eg, 95% confidence interval). Make clear which confounders were adjusted for and why they were included | |  |
|  |  | (*b*) Report category boundaries when continuous variables were categorized | |  |
|  |  | (*c*) If relevant, consider translating estimates of relative risk into absolute risk for a meaningful time period | |  |

| Other analyses | 17 | Report other analyses done—eg analyses of subgroups and interactions, and sensitivity analyses |  |
| --- | --- | --- | --- |
|  |  | **Discussion** |  |
| Key results | 18 | Summarise key results with reference to study objectives | 12,13,14,15 |
| Limitations | 19 | Discuss limitations of the study, taking into account sources of potential bias or imprecision. Discuss both direction and magnitude of any potential bias | 14 |
| Interpretation | 20 | Give a cautious overall interpretation of results considering objectives, limitations, multiplicity of analyses, results from similar studies, and other relevant evidence | 12,13,14 |
| Generalisability | 21 | Discuss the generalisability (external validity) of the study results | 15 |
| Other information | |  |  |
| Funding | 22 | Give the source of funding and the role of the funders for the present study and, if applicable, for the original study on which the present article is based | 16 |

**B. “Preferred Reporting Items for Systematic Reviews and Meta-Analyses” PRISMA Checklist**

| **Section and Topic** | **Item #** | **Checklist item** | **Location where item is reported** |
| --- | --- | --- | --- |
| **TITLE** | | |  |
| Title | 1 | Identify the report as a systematic review. | 1 |
| **ABSTRACT** | | |  |
| Abstract | 2 | See the PRISMA 2020 for Abstracts checklist. | 2 |
| **INTRODUCTION** | | |  |
| Rationale | 3 | Describe the rationale for the review in the context of existing knowledge. | 4 |
| Objectives | 4 | Provide an explicit statement of the objective(s) or question(s) the review addresses. | 5 |
| **METHODS** | | |  |
| Eligibility criteria | 5 | Specify the inclusion and exclusion criteria for the review and how studies were grouped for the syntheses. | 7-8 |
| Information sources | 6 | Specify all databases, registers, websites, organisations, reference lists and other sources searched or consulted to identify studies. Specify the date when each source was last searched or consulted. | 7-8 |
| Search strategy | 7 | Present the full search strategies for all databases, registers and websites, including any filters and limits used. | 7-8 |
| Selection process | 8 | Specify the methods used to decide whether a study met the inclusion criteria of the review, including how many reviewers screened each record and each report retrieved, whether they worked independently, and if applicable, details of automation tools used in the process. | 7-8 |
| Data collection process | 9 | Specify the methods used to collect data from reports, including how many reviewers collected data from each report, whether they worked independently, any processes for obtaining or confirming data from study investigators, and if applicable, details of automation tools used in the process. | 8 |
| Data items | 10a | List and define all outcomes for which data were sought. Specify whether all results that were compatible with each outcome domain in each study were sought (e.g. for all measures, time points, analyses), and if not, the methods used to decide which results to collect. | 9 |
|  | 10b | List and define all other variables for which data were sought (e.g. participant and intervention characteristics, funding sources). Describe any assumptions made about any missing or unclear information. | 8 |
| Study risk of bias assessment | 11 | Specify the methods used to assess risk of bias in the included studies, including details of the tool(s) used, how many reviewers assessed each study and whether they worked independently, and if applicable, details of automation tools used in the process. | 8 |
| Effect measures | 12 | Specify for each outcome the effect measure(s) (e.g. risk ratio, mean difference) used in the synthesis or presentation of results. | 9 |
| Synthesis methods | 13a | Describe the processes used to decide which studies were eligible for each synthesis (e.g. tabulating the study intervention characteristics and comparing against the planned groups for each synthesis (item #5)). | 8-9 |
|  | 13b | Describe any methods required to prepare the data for presentation or synthesis, such as handling of missing summary statistics, or data conversions. | 9-10 |
|  | 13c | Describe any methods used to tabulate or visually display results of individual studies and syntheses. | 9-10 |
|  | 13d | Describe any methods used to synthesize results and provide a rationale for the choice(s). If meta-analysis was performed, describe the model(s), method(s) to identify the presence and extent of statistical heterogeneity, and software package(s) used. | 9-10 |
|  | 13e | Describe any methods used to explore possible causes of heterogeneity among study results (e.g. subgroup analysis, meta-regression). | 9-10 |
|  | 13f | Describe any sensitivity analyses conducted to assess robustness of the synthesized results. | 9-10 |
| Reporting bias assessment | 14 | Describe any methods used to assess risk of bias due to missing results in a synthesis (arising from reporting biases). | 8 |
| Certainty assessment | 15 | Describe any methods used to assess certainty (or confidence) in the body of evidence for an outcome. | 8 |
| **RESULTS** | | |  |
| Study selection | 16a | Describe the results of the search and selection process, from the number of records identified in the search to the number of studies included in the review, ideally using a flow diagram. | 11 |
|  | 16b | Cite studies that might appear to meet the inclusion criteria, but which were excluded, and explain why they were excluded. | 11 |
| Study characteristics | 17 | Cite each included study and present its characteristics. | 11 |
| Risk of bias in studies | 18 | Present assessments of risk of bias for each included study. | 11 |
| Results of individual studies | 19 | For all outcomes, present, for each study: (a) summary statistics for each group (where appropriate) and (b) an effect estimate and its precision (e.g. confidence/credible interval), ideally using structured tables or plots. | 11-12 |
| Results of syntheses | 20a | For each synthesis, briefly summarise the characteristics and risk of bias among contributing studies. | 11-12 |
|  | 20b | Present results of all statistical syntheses conducted. If meta-analysis was done, present for each the summary estimate and its precision (e.g. confidence/credible interval) and measures of statistical heterogeneity. If comparing groups, describe the direction of the effect. | 11-12 |
|  | 20c | Present results of all investigations of possible causes of heterogeneity among study results. | 11-12 |
|  | 20d | Present results of all sensitivity analyses conducted to assess the robustness of the synthesized results. | 11-12 |
| Reporting biases | 21 | Present assessments of risk of bias due to missing results (arising from reporting biases) for each synthesis assessed. | 11-12 |
| Certainty of evidence | 22 | Present assessments of certainty (or confidence) in the body of evidence for each outcome assessed. | 11-12 |
| **DISCUSSION** | | |  |
| Discussion | 23a | Provide a general interpretation of the results in the context of other evidence. | 12-14 |
|  | 23b | Discuss any limitations of the evidence included in the review. | 14-15 |
|  | 23c | Discuss any limitations of the review processes used. | 14-15 |
|  | 23d | Discuss implications of the results for practice, policy, and future research. | 15 |
| **OTHER INFORMATION** | | |  |
| Registration and protocol | 24a | Provide registration information for the review, including register name and registration number, or state that the review was not registered. | 7 |
|  | 24b | Indicate where the review protocol can be accessed, or state that a protocol was not prepared. | 7 |
|  | 24c | Describe and explain any amendments to information provided at registration or in the protocol. | 7-8 |
| Support | 25 | Describe sources of financial or non-financial support for the review, and the role of the funders or sponsors in the review. | 16 |
| Competing interests | 26 | Declare any competing interests of review authors. | 16 |
| Availability of data, code and other materials | 27 | Report which of the following are publicly available and where they can be found: template data collection forms; data extracted from included studies; data used for all analyses; analytic code; any other materials used in the review. | 7 |

**C. Details on the risk of bias assessment**

Two authors (EX and NAX) independently assessed the risk of bias of included studies. Any disagreements were discussed with the corresponding author (IIS).

For observational studies, we used the Tool to Assess Risk of Bias in Cohort Studies, developed by the CLARITY Group at McMaster University. The tool uses 8 questions, with 4 possible answers in each. The tool divides the cohorts as exposed and non-exposed. The examples beneath the questions are intended to clarify the rationale behind answers in each question.

In each question, 4 answers were possible:

1.Definitely yes (low risk of bias)

2. Probably yes

3. Probably no

4. Definitely no (high risk of bias)

*Q1 Was selection of treated and control cohorts drawn from the same population?*

Definitely yes: Treated and control patients drawn from same administrative database of patients presenting at the same point of care, over the same time frame (<=3 months).

Probably yes: Treated and control patients drawn from same administrative database of patients presenting at the same point of care, over a similar time frame (>3 but <12 months).

Probably no:  Treated and control patients presenting to different points of care (e.g., multicenter study) or at the same point of care, over a different time frame (>= 12 months)

Definitely no: Treated and control patients presenting to unspecified points of care.

*Q2. Can we be confident in the presence of treated and control cohorts?*

Based on the inclusion criteria, a definitely yes was pre-specified as the appropriate answer.

*Q3. Can we be confident that the outcome of interest was not present at start of study?*

Since our main outcome of interest (mortality) could only occur after COVID-19 infection and treatment with vitamin C (or lack thereof), a definitely yes was pre-specified as the appropriate answer.

*Q4. Did the study match exposed and unexposed for all variables that are associated with the outcome of interest or did the statistical analysis adjust for these prognostic variables?*

Definitely yes: Matching or adjustment for all the prognostic variables on treated/control cohorts’ outcomes.

Probably yes: Matching or adjustment for some prognostic variables on treated/control  cohorts’ outcomes.

Probably no: Matching or adjustment for one prognostic variable on treated/control cohorts’ outcomes.

Definitely no: No matching or adjustments for prognostic variables on treated/control  cohorts’ outcomes.

*Q5. Can we be confident in the assessment of the presence or absence of prognostic factors?*

Definitely yes: Data collection on prognostic variables through electronic medical records.

Probably yes: Data collection through database or review of charts.

Probably no: Data collection without demonstration of reproducibility.

Definitely no: Data collection process not stated or no data on prognostic factors regarding treated/control cohorts.

*Q6. Can we be confident in the assessment of outcome?*

Due to the nature of our primary outcome (all-cause mortality), a definitely yes was pre-specified as the appropriate answer.

*Q7.Was the follow up of cohorts adequate?*

Definitely yes: Median follow up of at least 28 days, or all patients discharged or dead.

Probably yes: Median follow up between 14-28 days, or the majority of patients discharged or dead.

Probably no: Median follow-up between 7 and up to but not including 14 days.

Definitely no: Median follow-up less than 7 days or not stated.

*Q8. Were co-interventions similar between groups?*

Definitely yes: when it is explicitly stated that the co-interventions were similar between groups or one can derive from the data provided in the study that there was a high % of accordance (>= 80%) between the co-interventions of the two groups.

Probably yes: when treated and control patients were drawn from same administrative database of patients presenting at the same point of care, over the same time frame (<3months).

Probably no:  when treated and control patients were presenting to different points of care (e.g., multicenter study) or at the same point of care, over a similar or different time frame (>=3months), due to the accrual of knowledge regarding COVID-19 and the constant evolution of good clinical practice.

Definitely no: when treated and control patients presented to unspecified points of care.

For randomised controlled trials, we used Risk of Bias 2 (RoB2) assessment form was used^(2)^. The tool uses 5 domains with up to 7 questions each. In each question, 5 answers were possible:

1.Yes (low risk of bias)

2. Probably yes

3. Probably no

4. No

5. Not identified

After summarising all the results from each of the 5 domains, an overall risk was calculated, with 3 possible ratings:

1. High

2. Some concerns

3. Low

**D. e-Tables and e-Figures.**

| **e-Table 1. Risk of bias assessment of the included studies in the meta-analysis** | | | | | | | | |
| --- | --- | --- | --- | --- | --- | --- | --- | --- |
| Observational studies | | | | | | | | |
| Author | Q1 | Q2 | Q3 | Q4 | Q5 | Q6 | Q7 | Q8 |
| Gao | 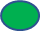 | 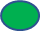 | 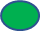 | 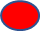 | 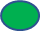 | 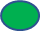 | 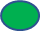 | 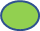 |
| Gavrielatou | 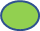 | 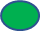 | 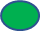 | 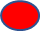 | 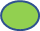 | 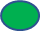 | 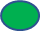 | 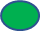 |
| Li | 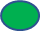 | 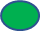 | 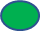 | 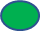 | 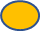 | 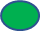 | 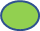 | 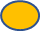 |
| Krishnan | 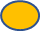 | 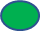 | 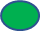 | 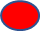 | 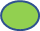 | 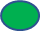 | 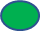 | 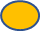 |
| Sulaiman | 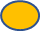 | 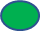 | 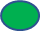 | 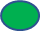 | 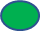 | 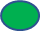 | 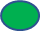 | 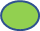 |
| Zheng | 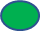 | 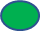 | 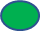 | 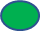 | 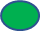 | 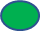 | 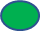 | 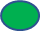 |
| Randomized controlled trials | | | | | | | | |
| Author | D1 | D2 | D3 | D4 | D5 | Overall | | |
| Beigmohammadi | 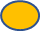 | 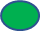 | 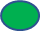 | 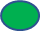 | 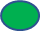 | 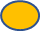 | | |
| Darban | 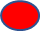 | 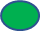 | 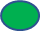 | 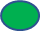 | 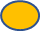 | 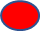 | | |
| JamaliMoghadamSiahkali | 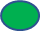 | 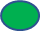 | 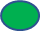 | 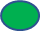 | 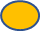 | 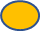 | | |
| Kumari | 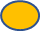 | 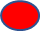 | 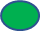 | 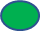 | 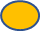 | 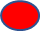 | | |
| Zhang | 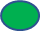 | 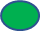 | 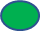 | 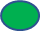 | 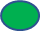 | 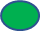 | | |

Abbreviations:

Q=Question;
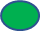
= Definitely Yes;
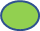
=Probably Yes;
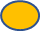
=Probably No;
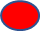
=Definitely No

 D=Domain;
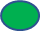
= Low;
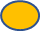
= Some concerns;
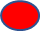
= High

**e-Figure 1.** All-cause mortality of critically ill patients with COVID-19 receiving vitamin C on top of standard-of-care (vitamin C group) versus standard-of-care alone (control group) in the sensitivity analysis of studies with low risk of bias. Pooled risk ratio (RR) and 95% confidence intervals (CI) were calculated using a random effects model.

**e-Figure 2.** All-cause mortality of critically ill patients with COVID-19 receiving vitamin C on top of standard-of-care (vitamin C group) versus standard-of-care alone (control group) in the sensitivity analysis of randomized controlled trials. Pooled risk ratio (RR) and 95% confidence intervals (CI) were calculated using a random effects model.

**e-Figure 3.** Length of stay in the intensive care unit of critically ill patients with COVID-19 receiving vitamin C on top of standard-of-care (vitamin C group) versus standard-of-care alone (control group). Mean difference (MD) and 95% confidence intervals (CI) were calculated using a random effects model.

**e-Figure 4.** Duration of mechanical ventilation of critically ill patients with COVID-19 receiving vitamin C on top of standard-of-care (vitamin C group) versus standard-of-care alone (control group). Mean difference (MD) and 95% confidence intervals (CI) were calculated using a random effects model.


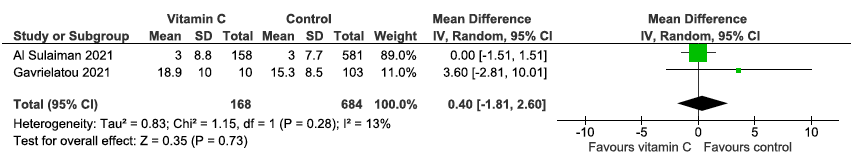


**e-Figure 5.** Need for renal replacement therapy of critically ill patients with COVID-19 receiving vitamin C on top of standard-of-care (vitamin C group) versus standard-of-care alone (control group). Pooled risk ratio (RR) and 95% confidence intervals (CI) were calculated using a random effects model.


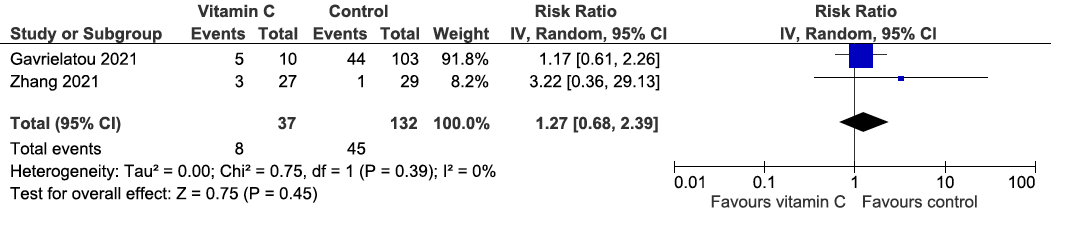

Supplement: Supplementary file 1 [file Data_Sheet_1.docx]
